# Genetic and biochemical characterization of OXA-1054, a carbapenem-hydrolyzing class D β-lactamase conferring broad-spectrum β-lactam resistance in *Pseudomonas aeruginosa*

**DOI:** 10.1128/aac.01805-25

**Published:** 2026-05-29

**Authors:** Lucía González-Pinto, Laura Monge-Olivares, Gloria Pérez-Rodríguez, Pablo Aja-Macaya, Lucía Sánchez-Peña, María Antonia Gomis-Font, Salud Rodríguez-Pallares, Tania Blanco-Martín, Lorena López-Cerero, Alejandro Beceiro, Pierre Bogaerts, Antonio Oliver, Germán Bou, Jorge Arca-Suárez

**Affiliations:** 1 Servicio de Microbiología Clínica & Grupo de Investigación en Microbiología. Instituto de Investigación Biomédica de A Coruña (INIBIC), Complexo Hospitalario Universitario de A Coruña (CHUAC), SERGAS, Universidade da Coruña (UDC), A Coruña, Spain; 2 CIBER de Enfermedades Infecciosas (CIBERINFEC), Instituto de Salud Carlos III 38176, Madrid, España; 3 Instituto de Biomedicina de Sevilla IBIS, Hospital Universitario Virgen Macarena/CSIC/Universidad de Sevilla 16778, Seville, Spain; 4 Departamento de Microbiología, Facultad de Medicina, Universidad de Sevilla 16778, Seville, Spain; 5 Servicio de Microbiología, Servicio de Microbiología and Unidad de Investigación, Hospital Universitario Son Espases, Instituto de Investigación Sanitaria Illes Balears (IdISBa) 375118, Palma de Mallorca, Spain; 6 Unidad de Enfermedades Infecciosas y Microbiología Clínica, Hospital Universitario Virgen Macarena 16582, Seville, Spain; 7 National Reference Laboratory for Monitoring of Antimicrobial Resistance in Gram-Negative Bacteria, CHU Dinant-Godinne, UCL Namur 82470 , Yvoir, Belgium; 8 Departamento de Fisioterapia, Medicina y Ciencias Biomédicas, Universidad de A Coruña 16737 , A Coruña, Spain; University of Fribourg, Fribourg, Switzerland

**Keywords:** β-lactam/β-lactamase inhibitor combination resistance, carbapenem resistance, β-lactam resistance, carbapenem-resistant *Pseudomonas aeruginosa*, carbapenemase-producing *Pseudomonas aeruginosa*, class D carbapenemases

## Abstract

We aimed to characterize OXA-1054, a novel carbapenem-hydrolyzing class D β-lactamase (CHDL) detected in a multidrug-resistant *Pseudomonas aeruginosa* clinical isolate. Antimicrobial susceptibility was determined by broth microdilution, and carbapenemase activity was confirmed by hydrolysis assays. Whole-genome sequencing via Illumina and PacBio platforms enabled comprehensive analysis of the resistome and plasmid architecture. The *bla*_OXA-1054_ gene was cloned in parallel with *bla*_OXA-48_ and *bla*_OXA-198_ into the pUCP24 plasmid. β-Lactamases were produced in *P. aeruginosa* PAO1 for comparative evaluation of the phenotypic impact of each. OXA-48, OXA-198, and OXA-1054 β-lactamases were purified and further subjected to steady-state kinetic analysis, and 50% inhibitory activity of β-lactamase inhibitors was determined. The clinical *P. aeruginosa* isolate ARGA00461 showed resistance to carbapenems and most β-lactam/β-lactamase inhibitor combinations and tested positive in carbapenem hydrolysis assays. Genomic analysis revealed that the *P. aeruginosa* isolate ARGA00461 carried a gene coding for a previously uncharacterized CHDL, designated OXA-1054, which is closely related to the OXA-372 enzyme of environmental origin. The *bla*_OXA-1054_ gene was located in a small (≈5 kbp) non-conjugative plasmid coexisting with a conjugative IncP plasmid, which likely facilitated its mobilization. Production of OXA-1054 in *P. aeruginosa* PAO1 conferred a broader spectrum of β-lactam resistance than other CHDLs. Enzyme kinetics confirmed carbapenem hydrolysis with catalytic efficiency comparable to OXA-48 and revealed higher affinity for cefepime compared to OXA-48 and OXA-198. Among the inhibitors tested, only avibactam demonstrated relevant inhibitory activity. OXA-1054 mediates broad-spectrum β-lactam resistance in *P. aeruginosa*, including carbapenems and β-lactam/β-lactamase inhibitor combinations.

## INTRODUCTION

*Pseudomonas aeruginosa* is an opportunistic pathogen frequently implicated in life-threatening healthcare-associated infections, such as ventilator-associated pneumonia and bloodstream infections, particularly among critically ill patients in intensive care units (1). One of the major challenges in managing *P. aeruginosa* infections is the remarkable ability of the pathogen to develop resistance to almost all clinically available β-lactam agents, including carbapenems, still considered the cornerstone of antipseudomonal therapy (2). Consequently, carbapenem-resistant *P. aeruginosa* (CRPA) has been classified by the World Health Organization as a high-priority pathogen with limited treatment options (3). Carbapenem resistance in *P. aeruginosa* is primarily driven by inactivating mutations in the outer membrane porin OprD, typically conferring resistance to imipenem and reduced susceptibility to meropenem. The accumulation of additional mutations affecting *ampC* gene expression and/or efflux pump activity results in high-level carbapenem resistance (4). Beyond mutational mechanisms, carbapenem resistance in *P. aeruginosa* is increasingly associated with the horizontal acquisition of carbapenemase-encoding genes. Carbapenemase production in *P. aeruginosa* is often linked to specific lineages with difficult-to-treat resistance phenotypes, known as high-risk clones, which have become globally disseminated in hospital settings (5). Infections caused by carbapenemase-producing CRPA are associated with significantly higher mortality rates than those caused by non-carbapenemase producers (6). Of particular concern, the activity of cefiderocol and other recently introduced β-lactam/β-lactamase inhibitor combinations are often limited against these carbapenemase producers (7). Metallo-β-lactamases (MBLs), such as VIM- and IMP-type enzymes, have historically been the predominant carbapenemases detected in *P. aeruginosa*. In recent years, however, carbapenemases more typically associated with Enterobacterales, including GES, KPC, and NDM enzymes, have increasingly been reported in *P. aeruginosa* isolates worldwide. According to National Center for Biotechnology Information (NCBI) databases, *bla*_KPC_ and *bla*_NDM_-type carbapenemases are now among the top five horizontally acquired carbapenemase genes identified in available *P. aeruginosa* genomes (8).

In contrast to class A and B carbapenemases, detection of class D carbapenem-hydrolyzing enzymes (CHDLs) in *P. aeruginosa* has been rarely reported. CHDLs are major contributors to carbapenem resistance in *Acinetobacter baumannii*, in which OXA-23, OXA-24/40, and OXA-58 are widespread (9). CHDLs are also highly prevalent in Enterobacterales, in which OXA-48-like enzymes have become extensively disseminated across species via highly promiscuous IncL/M plasmids (10). CHDLs are characterized by conferring carbapenem resistance, poor hydrolytic activity against third- and fourth-generation cephalosporins, and resistance to most clinically available β-lactamase inhibitors (11). A novel group of class D carbapenemases, designated OXA-198, was previously identified in IncP-type plasmids in clonally related *P. aeruginosa* isolates from Belgian hospitals, with no further reports to date (12).

Here, we sought to characterize OXA-1054, a novel plasmid-encoded class D carbapenemase involved in resistance to carbapenems and to recently developed β-lactam/β-lactamase inhibitor combinations in a clinical isolate of *P. aeruginosa* from Spain.

## RESULTS AND DISCUSSION

### Antimicrobial susceptibility of the clinical isolate *P. aeruginosa* ARGA00461

Antimicrobial susceptibility testing (Table 1) showed that the *P. aeruginosa* isolate ARGA00461 was resistant to piperacillin/tazobactam, ceftazidime, ceftolozane/tazobactam, cefepime, cefepime/taniborbactam, cefepime/zidebactam, imipenem, imipenem/relebactam, meropenem, and meropenem/vaborbactam. However, it remained susceptible to aztreonam, ceftazidime/avibactam, cefiderocol, aminoglycosides, quinolones, and colistin. This multidrug-resistant antimicrobial susceptibility profile was unexpected in a patient with no prior intensive care unit (ICU) admission, previously documented *P. aeruginosa* infections, or exposure to broad-spectrum β-lactams, which are risk factors for CRPA infections (13). In Spain, most circulating CRPA isolates usually remain susceptible to ceftolozane/tazobactam and imipenem/relebactam, as resistance in these strains is typically mutation-driven and not associated with carbapenemase production, which limit the activity of these agents. The most recent nationwide *P. aeruginosa* surveillance study (2022) reported 97.8% susceptibility to ceftolozane/tazobactam, with fewer than 2.1% of isolates carrying carbapenemases (5). In line with these observations, cloxacillin inhibition assays yielded a negative result, while the β-carba test and the CIM test yielded positive results, suggesting that the isolate was producing a carbapenem-hydrolyzing enzyme (data not shown). Immunochromatographic assays and PCR-based tests targeting the main carbapenemase families (OXA-48, KPC, NDM, VIM, and IMP) were consistently negative, indicating that the strain may be producing a different type of carbapenemase.

**TABLE 1:** Antimicrobial susceptibility profile and mechanisms of β-lactam resistance in the *P. aeruginosa* ARGA00461 clinical isolate carrying *bla*_OXA-1054_

Strain ID	ST	MIC (mg/L)^*a*^^,*b*^																			β-lactam resistance mechanisms	
		TIC (R>16)	P/T (R>16)	AZT (R>16)	A/A (R>16)	CAZ (R>8)	C/A (R>8)	C/T (R>4)	FEP (R>8)	F/T (R>8)	F/Z (R>8)	FDC (R>2)	IMP (R>4)	I/R (R>2)	MEM (R>8)	M/V (R>8)	TOB (R>2)	AK (R>16)	CIP (R>0.5)	COL (R>4)	Transferable	Mutational
**ARGA00461**	303	>512	256	16	16	16	8	64	128	64	16	1	64	64	64	64	≤2	≤8	≤0.05	≤2	*bla*_OXA-1054_	∆*nalC*

^*a*^ TIC: ticarcillin; P/T: piperacillin/tazobactam; AZT: aztreonam; A/A: aztreonam/avibactam; CAZ: ceftazidime; C/A: ceftazidime/avibactam; C/T: ceftolozane/tazobactam; FEP: cefepime; F/T: cefepime/taniborbactam; F/Z: cefepime/zidebactam; FDC: cefiderocol; IMP: imipenem; I/R: imipenem/relebactam; MEM: meropenem; M/V: meropenem/vaborbactam; TOB: tobramycin; AK: amikacin; CIP: ciprofloxacin; COL: colistin.

^*b*^ EUCAST v. 15.0 breakpoints for *Pseudomonas* spp. indicated. For combinations not yet approved, the breakpoint of the β-lactam alone is indicated.

### Genetic features of a new class D β-lactamase, OXA-1054, and resistome of *P. aeruginosa* isolate ARGA00461

The isolate was genetically characterized by short- and long-read sequencing. According to PubMLST databases, *P. aeruginosa* isolate ARGA00461 was assigned to sequence type 303. This lineage has been linked to both nosocomial and community-acquired infections (14). Analysis of horizontally acquired resistance determinants detected the presence of a previously uncharacterized 257 amino-acid length class D carbapenemase, which was designated OXA-1054. OXA-1054 belongs to OXA-372-like carbapenemases, sharing 93.8% amino acid identity and 16 amino acid substitutions relative to the parental OXA-372. The OXA-372 carbapenemase was previously identified in a *Citrobacter freundii* environmental isolate recovered from a hospital wastewater plant in central Italy, and it defined a new type of class D CHDLs (15). Like OXA-372, OXA-1054 was distantly related to other class D carbapenemases, such as OXA-198 (55.8% similarity), OXA-48 (40.4% similarity), and OXA-24 (27.9% similarity). To date, three variants belonging to the OXA-372 family have been identified: OXA-372, OXA-641, and OXA-1016. OXA-641 was detected in carbapenem-resistant clinical isolates of *Morganella morganii* included in a comparative genomic study conducted in France (16). OXA-1016 was found in an *Ectopseudomonas oleovorans* clinical isolate identified in the same country (GenBank accession number: NG_076676.1). Finally, the *bla*_OXA-1054_ gene identified in this clinical *P. aeruginosa* isolate was previously identified by our group in Enterobacterales species recovered from environmental samples collected from wastewater treatment plants in Seville (South Spain) (17). However, beyond the parental OXA-372, the genetic characteristics, impact on β-lactam resistance, and biochemical properties of other OXA-372-like β-lactamases have not yet been characterized.

**Fig 1:**
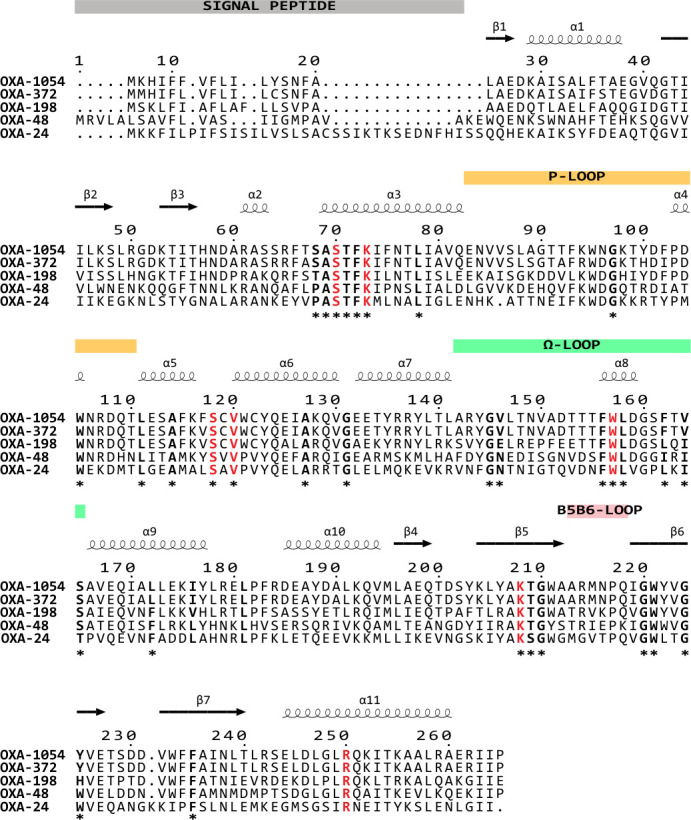
Sequence alignment of OXA-1054, OXA-372, OXA-198, OXA-48 (the reference sequence), and OXA-24 β-lactamases. The signal peptide, which is removed post-translationally during export to the bacterial periplasm, was assigned using Deepsig (21) and indicated in gray. Loops framing the active site are mapped above the corresponding residue numbers. Residues involved in catalytic activity are highlighted in red. Positions identified to be highly conserved based on the structural alignment-based numbering of class D β-lactamases (18) are indicated by an asterisk.

Figure 1 shows the sequence alignment of OXA-1054 and other CHDLs of high clinical relevance following the recently developed SAND alignment scheme (structural alignment-based numbering of class D β-lactamases) (18) and using the OXA-48 sequence as the reference. The CHDLs are displayed in order of amino acid identity: OXA-372, OXA-198, OXA-48, and OXA-24. Comparative sequence analysis revealed that OXA-372 shares the catalytically important amino acid arrangement and motifs required for the enzyme activity with the other class D carbapenemases included in the analysis: S-T-F-K in position 70, S-X-V in position 118, Y/F-G-V/E/N in position 144, W in position 157, KTG in position 208, and R in position 250. Regarding the differences relative to OXA-372, most occur within the P-loop, a conserved structural motif located near the active site of the enzyme and which plays a key role in substrate recognition, catalytic activity, and structural stability, and also, importantly, a V117F change adjacent to the S-X-V motif (19, 20).

Beyond the horizontally acquired *bla*_OXA-1054_ gene, comparative analysis of the resistome of *P. aeruginosa* isolate ARGA00461 and that of the naturally susceptible *P. aeruginosa* PAO1 strain as reference only revealed inactivation of the *nalC* gene caused by the insertion of a phage. Inactivation of the *nalC* regulator drives hyperproduction of the MexAB-OprM efflux pump. Overproduction of MexAB-OprM has important effects on the activity of several β-lactams in *P. aeruginosa*, such as aztreonam, ceftazidime, and meropenem (22). The substrate profile of MexAB-OprM also includes newly approved or investigational β-lactamase inhibitors, such as relebactam, zidebactam, and xeruborbactam (23). This suggests that overproduction of MexAB-OprM contributes to β-lactam resistance in *P. aeruginosa* isolate ARGA00461.

### Genetic context of *bla*_OXA-1054_

**Fig 2:**
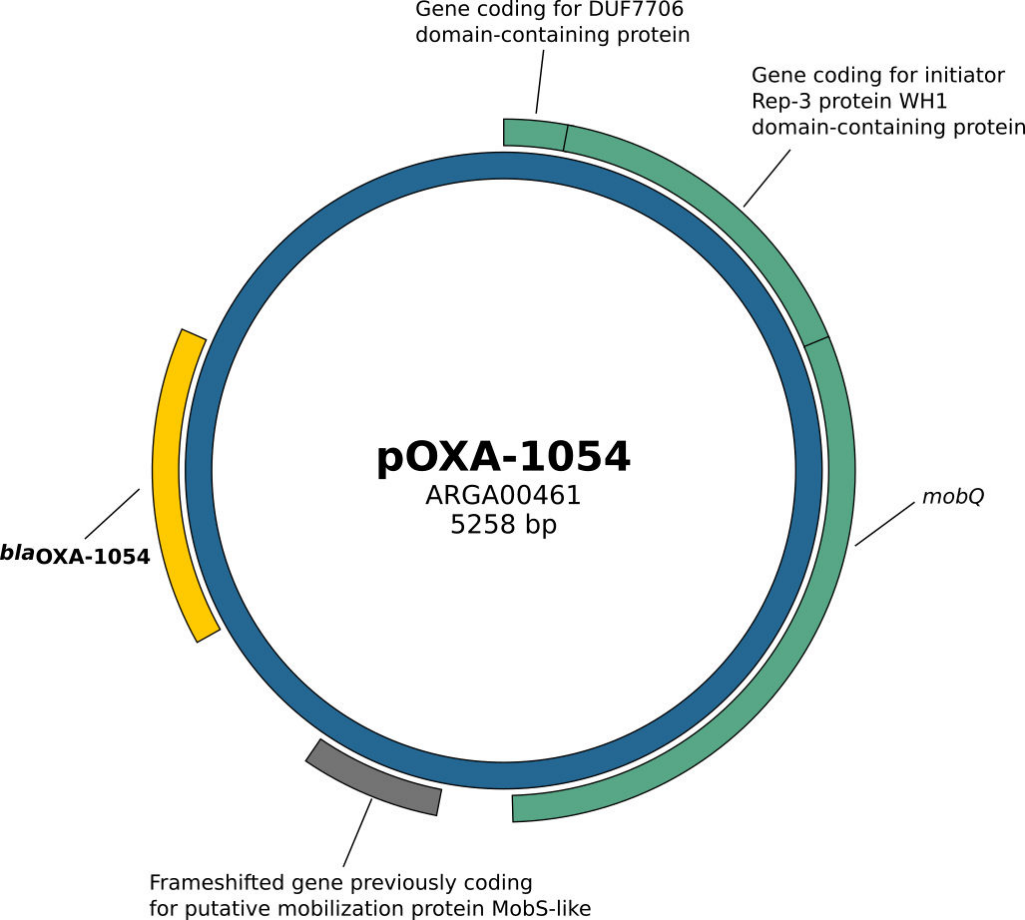
Genetic context of the pOXA-1054 plasmid carrying the *bla*_OXA-1054_ gene in *P. aeruginosa* isolate ARGA00461. Genes are colored green. Frameshifted genes are colored gray. The gene of interest, *bla*_OXA-1054_, is highlighted in yellow.

Long-read sequencing enabled complete circularization of the mobile genetic elements present in *P. aeruginosa* isolate ARGA00461, revealing two different plasmids. The *bla*_OXA-1054_ gene was located on a 5258-bp plasmid (designated pOXA-1054), which did not carry additional resistance determinants (Fig. 2). No incompatibility group could be assigned to pOXA-1054 using public databases, although its replication initiator protein allegedly belongs to the Rep-3 family, suggesting that it represents a currently untyped small plasmid replicon. No insertion sequences, transposases, or other recognizable mobilization elements were detected in pOXA-1054. BLAST analysis showed that this plasmid shared 100% sequence identity and 83% coverage with a plasmid previously detected in an environmental microorganism belonging to the species *Candidatus Nitrotoga fabula* (GenBank accession number: LS423453.1), a nitrite-oxidizing bacteria frequently found in wastewater treatment plants (24), in which the *bla*_OXA-1054_ gene was absent. In particular, the locus harboring the *bla*_OXA-1054_ carbapenemase gene in pOXA-1054 is replaced in the *Candidatus Nitrotoga fabula* plasmid by a Qac-pB8 family SMR efflux transporter, which confers resistance to quaternary ammonium compounds commonly found in wastewater environments, and a TetR-family transcriptional regulator that likely controls the expression of the efflux pump. A comparison between both plasmids is shown in Fig. S1. Such origin in environmental bacterial species, as well as the circulation of the gene in Enterobacterales species recovered from wastewater treatment plants observed in our previous work (17), reinforces the following: (i) the role of environmental bacteria as the source of antimicrobial resistance determinants, like OXA-1054, which may ultimately end in clinical isolates driving life-threatening infections in humans; and (ii) the potential of this carbapenemase to circulate in both Enterobacterales and *P. aeruginosa* backgrounds.

Plasmid pOXA-1054 lacked the genetic machinery required for conjugation or transference and was, therefore, predicted to be non-conjugative by the MOB-suite tool. Notably, *P. aeruginosa* strain ARGA00461 also carried a 126-kb plasmid, designated pAC880 by MOB-suite (Fig. S2), classified within the IncP incompatibility group. pAC880 carries a complete mercury resistance (*mer*) operon, which could be involved in detoxification of inorganic mercury. It also encodes an ABC transporter and a HicA toxin-antitoxin system, which may contribute to stress tolerance and persistence phenotypes, as well as a carbon storage regulator (CsrA) potentially involved in metabolic regulation and biofilm-associated traits, but showed no additional determinants for β-lactam resistance. We identified highly similar plasmids (>98% identity and >85% coverage) mainly in *P. aeruginosa* isolates of clinical origin, although available sequences are limited. We also identified related plasmid backbones in other environmental *Pseudomonas* species, including *P. putida*, *P. veronii*, and *P. fluorescens*, and more sporadically in *Stutzerimonas* spp. and *Pandoraea pnomenusa*, suggesting that pAC880-like plasmids may circulate across clinical and environmental bacterial populations. According to the MOB-suite tool, the pAC880 plasmid is a conjugative plasmid able to self-transfer to other bacterial cells, as predicted by the presence of a complete set of genes associated with plasmid transfer, including *tra*-family genes. The coexistence of both a conjugative IncP plasmid (pAC880) and a small non-conjugative plasmid (pOXA-1054) within the same strains suggests that pOXA-1054 takes advantage of the conjugation machinery encoded in the accompanying pAC880 plasmid to spread, as previously described for other CHDL-encoding genes like OXA-24 (25). Thus, we explored whether the *bla*_OXA-1054_ gene could be transferred to other bacteria through conjugation assays. Our results demonstrate that pOXA-1054 was successfully transferred from *P. aeruginosa* ARGA00461 to the rifampicin-resistant *P. aeruginosa* PAO1^Rif^ strain with a conjugation efficiency of 3.6 × 10^−6^. These findings confirm that pAC880 facilitates the mobilization of the non-conjugative plasmid pOXA-1054 to other clinical strains, likely playing a key role in the dissemination of this carbapenemase.

### Impact of OXA-1054 production on β-lactam resistance in *P. aeruginosa* and detection of carbapenemase activity

The antimicrobial susceptibility data of the recombinant *P. aeruginosa* PAO1 strains producing OXA-1054 in both the pUCP24 laboratory plasmid as well as the pOXA-1054 natural plasmid are shown in Table 2. To better understand the impact of OXA-1054 production on β-lactam resistance, the β-lactamases OXA-48 and OXA-198 were also produced in *P. aeruginosa* PAO1 for comparative purposes. In line with observations for the clinical isolate *P. aeruginosa* ARGA00461, the *P. aeruginosa* PAO1 transformants producing OXA-1054 showed significant MIC increases to piperacillin/tazobactam, ceftazidime, ceftolozane/tazobactam, cefepime, cefepime/taniborbactam, cefepime/zidebactam, cefiderocol, imipenem/relebactam, meropenem, and meropenem/vaborbactam. These results support the broad-spectrum of β-lactam resistance conferred by OXA-1054 in the *P. aeruginosa* background. The MICs of aztreonam, aztreonam/avibactam, ceftazidime/avibactam, and imipenem did not increase significantly. Although cloning experiments reproduced the major resistance phenotype associated with OXA-1054, some differences in aztreonam and imipenem MIC values compared with the clinical isolate suggest additional strain-specific factors or regulatory mechanisms that may also contribute to the β-lactam resistance phenotype.

**TABLE 2:** Antimicrobial susceptibility data for recombinant *P. aeruginosa* PAO1 isolates producing OXA-48, OXA-198, and OXA-1054 β-lactamases and results of carbapenem hydrolysis detection tests

Strain	MIC (mg/L)^*a*^^,*b*^														Carbapenem hydrolysis detection		
	P/T (R>16*)*	AZT (R>16)	A/A (R>16)	CAZ (R>8)	C/A (R>8)	C/T (R>4)	FEP (R>8)	F/T (R>8)	F/Z (R>8)	FDC (R>2)	IMP (R>4)	I/R (R>2)	MEM (R>8)	M/V (R>8)	Cloxacillin inhibition test	CIMtest	β-Carba test
PAO1	4	4	2	2	1	0.5	2	2	1	0.125	1	0.25	1	0.5	−	−	−
PAO1 + OXA-48	256	4	2	2	2	2	16	2	2	0.125	16	8	64	64	−	+	+
PAO1 + OXA-198	64	2	2	2	1	8	4	2	2	0.25	2	2	2	2	−	+	+
PAO1 + OXA-1054	256	8	2	16	2	32	64	16	4	2	4	4	32	32	−	+	+
PAO1 + pOXA-1054^*c*^	256	16	4	8	2	32	64	8	4	2	16	16	32	32	−	+	+

^*a*^ P/T: piperacillin/tazobactam; AZT: aztreonam; A/A: aztreonam/avibactam; CAZ: ceftazidime; C/A: ceftazidime/avibactam; C/T: ceftolozane/tazobactam; FEP: cefepime; F/T: cefepime/taniborbactam; F/Z: cefepime/zidebactam; FDC: cefiderocol; IMP: imipenem; I/R: imipenem/relebactam; MEM: meropenem; M/V: meropenem/vaborbactam.

^*b*^ EUCAST v. 15.0 breakpoints for *Pseudomonas* spp. indicated. For combinations not yet approved, the breakpoint of the β-lactam alone is indicated.

^*c*^ Recombinant *P. aeruginosa *PAO1 transformant carrying clinical plasmid with the* bla*_OXA-1054_.

The most remarkable feature of OXA-1054 relative to other OXA-48 or OXA-198 enzymes, which hydrolyze cephalosporins very poorly (26), is its important impact on the activity of ceftazidime, ceftolozane/tazobactam, cefepime/taniborbactam, cefepime/zidebactam, and cefiderocol. This phenotype reflects a broad substrate profile beyond that of other class D enzymes, with a particularly pronounced effect on cephalosporins, including ceftazidime, cefepime, and ceftolozane/tazobactam. Notably, addition of avibactam restored ceftazidime susceptibility in the ceftazidime-resistant *P. aeruginosa* PAO1 OXA-1054 transformant, suggesting that this enzyme is susceptible to avibactam inhibition. Taniborbactam and zidebactam slightly reversed the increase in cefepime MICs observed in the PAO1 OXA-1054-producing transformant. In contrast, addition of relebactam or vaborbactam did not reduce the MICs of imipenem or meropenem against any of the transformants producing CHDLs, which ineffectively block class D enzymes (27). Altogether, these data reveal a particular β-lactam substrate profile and susceptibility to β-lactamase inhibitors for this newly described carbapenemase. Comparative analysis of carbapenemase detection by hydrolysis assays revealed that all the CHDLs evaluated, including OXA-1054, were well detected. These findings indicate that hydrolysis methods are useful for the rapid detection of CHDLs in *P. aeruginosa*.

### Biochemical characteristics of OXA-1054

To characterize the biochemical properties of OXA-1054, the carbapenemase was purified in parallel with OXA-48 and OXA-198, and a comparative evaluation of the steady-state kinetics was conducted. The *K*_m_, *k*_cat_, and *k*_cat_/*K*_m_ values defining the β-lactam substrate profiles of each enzyme are summarized in Table 3. The kinetic parameters for meropenem and imipenem confirmed the carbapenem-hydrolyzing activity of OXA-1054. Imipenem proved to be the preferred carbapenem substrate for OXA-1054 (*k*_cat_/*K*_m_ = 0.51 µM^−1^ · s^−1^), whereas meropenem-hydrolyzing activity was about 10 times lower (*k*_cat_/*K*_m_ = 0.05 µM^−1^ · s^−1^). Although OXA-1054 *K*_m_ values for meropenem are 13.8 times lower than imipenem, the turnover rates for the latter are 117.2-fold higher, thus explaining the differences in hydrolysis. Carbapenem hydrolysis constants were very similar to that observed for OXA-48, with a *k*_cat_/*K*_m_ value of 0.312 µM · s^−1^. These values are almost identical to those reported by Docquier *et al*. (28). Compared with OXA-198, both OXA-1054 and OXA-48 showed lower carbapenem catalytic efficiencies, a phenomenon mainly driven by the very low *K*_m_ values noted for OXA-198 with meropenem and imipenem. These findings are consistent with those reported by El Garch *et al*., who described very low *K*_m_ and *k*_cat_ values for both imipenem and meropenem in the OXA-198 β-lactamase (12). Compared to OXA-372 carbapenemase (unfortunately not available for direct comparison), Antonelli *et al.* reported *k*_cat_/*K*_m_ values of 0.22 µM^−1^ · s^−1^ for imipenem and 0.52 µM^−1^ · s^−1^ for meropenem (15). OXA-1054 shows similar activity against imipenem but lower activity against meropenem. The differences in meropenem catalytic efficiency could be explained by the following: (i) differences in the methodology for monitoring meropenem hydrolysis, which the authors performed using benzylpenicillin as the reporter substrate; and (ii) the possible importance of 16-amino acid differences between OXA-1054 and OXA-372 for meropenem catalysis.

**TABLE 3:** Steady-state kinetic parameters of OXA-48, OXA-198, and OXA-1054 β-lactamases for representative substrates^*a*^

Substrate	*k*_cat_ (s^−1^)			*K*_m_ (μM)			*k*_cat_/*K*_m_ (μM^−1^ · s^−1^)		
	OXA-48	OXA-198	OXA-1054	OXA-48	OXA-198	OXA-1054	OXA-48	OXA-198	OXA-1054
Meropenem	0.07	0.01	0.03	2.56	0.05	0.58	0.03	0.20	0.05
Imipenem	2.93	0.57	3.49	9.55	0.16	6.97	0.31	3.66	0.51
Ceftolozane	ND	ND	ND	>2,000	>2,000	>2,000	ND	ND	ND
Ceftazidime	ND	ND	ND	>2,000	>2,000	>2,000	ND	ND	ND
Cefepime	ND	ND	ND	>2,000	>2,000	948.74	ND	ND	ND

^*a*^ ND: not determined. For cephalosporins, high *K*_m_ values prevented achievement of saturating substrate concentrations, precluding reliable determination of *k*_cat_ and *k*_cat_/*K*_m_ ratios. Standard deviations were below 30% of the mean for each parameter.

We also evaluated ceftolozane, ceftazidime, and cefepime as substrates in steady-state kinetic assays to investigate the enhanced ability of OXA-1054 to confer resistance to cephalosporins and β-lactam/β-lactamase inhibitor combinations when produced in *P. aeruginosa*. It was not possible to determine the *k*_cat_/*K*_m_ values for OXA-48, OXA-198, and OXA-1054 with any of the cephalosporin substrates tested due to the inability to reach saturating substrate concentrations under the experimental conditions. However, *K*_m_ values for cefepime could be estimated from competitive inhibition experiments for OXA-1054, revealing a lower *K*_m_ value for this variant compared with OXA-48 and OXA-198. This indicates a higher apparent affinity of OXA-1054 for cefepime, which could explain the increased cefepime resistance phenotype observed when OXA-1054 is produced in the complex *P. aeruginosa* background.

### Relative IC_50_ values of β-lactamase inhibitors against OXA-1054

We evaluated the inhibitory profiles of β-lactamase inhibitors tazobactam, avibactam, taniborbactam, zidebactam, relebactam, and vaborbactam, against OXA-1054 using OXA-48 and OXA-198 as comparator enzymes. The IC_50_ values calculated for these inhibitors are summarized in Table 4. OXA-1054 exhibited a particular susceptibility profile to β-lactamase inhibitors. Avibactam was the most potent inhibitor, blocking OXA-1054 activity in the nanomolar range. These observations are consistent with the phenotypic data obtained in the OXA-1054 *P. aeruginosa* PAO1 transformant, in which avibactam restored ceftazidime MICs to wild-type levels. On the contrary, zidebactam and taniborbactam displayed reduced inhibitory activity against OXA-1054 compared to avibactam, whereas vaborbactam, tazobactam, and relebactam lacked significant inhibitory activity. This β-lactamase inhibitor susceptibility profile was almost identical to that of OXA-198, with only avibactam IC_50_ values also in the nanomolar range. OXA-48 was susceptible to avibactam and also to taniborbactam, as recently described by our group (29). Similar observations were also made by Papp-Wallace *et al*. when evaluating the cefepime/taniborbactam combination against a large collection of OXA-48 producers (30). Altogether, the observations confirm the difficult-to-inhibit profile of these CHDLs, which proved to be resistant to most β-lactamase inhibitors, thus supporting continued drug development efforts.

**TABLE 4:** Determination of the 50% inhibitory concentrations of β-lactamase inhibitors for OXA-48, OXA-198, and OXA-1054 β-lactamases

β-Lactamase inhibitors		IC_50_ (μM)	
	OXA-48	OXA-198	OXA-1054
Tazobactam	1.49	260.09	>1,000
Avibactam	0.33	0.61	0.62
Relebactam	91.77	126.41	82.94
Zidebactam	10.88	7.12	9.55
Vaborbactam	28.41	>1,000	>1,000
Taniborbactam	0.08	24.44	35.50

## CONCLUSIONS

This study characterizes OXA-1054, a novel plasmid-encoded CHDL of the OXA-372 family, in an MDR *P. aeruginosa* clinical isolate from Spain. OXA-1054 confers broad β-lactam resistance in *P. aeruginosa*, including carbapenems, cephalosporins, and most β-lactam/β-lactamase inhibitor combinations, except ceftazidime/avibactam. Biochemical analyses confirmed carbapenemase activity and revealed limited cephalosporin hydrolysis but increased affinity for ceftazidime and cefepime relative to OXA-48 and OXA-198, likely contributing to the cephalosporin-resistant phenotype when produced in *P. aeruginosa*. The likely environmental origin of *bla*_OXA-1054_ and its associated plasmid highlights the role of environmental reservoirs in the emergence and dissemination of novel carbapenemases to clinical *P. aeruginosa* isolates. These findings expand the currently limited knowledge of CHDL diversity in *P. aeruginosa* and underscore the importance of sustained surveillance for emerging β-lactam resistance mechanisms in this continuously evolving pathogen.

## MATERIALS AND METHODS

### Clinical strains

The clinical *P. aeruginosa* strain ARGA00461 was isolated on 23 September 2024 (day 73 of hospitalization) from a rectal swab taken from a 56-year-old male patient who had been admitted to the ICU of the A Coruña University Hospital Complex with hemodynamic instability, requiring mechanical circulatory support and vasopressor therapy. No recent history of travel to foreign countries was documented for the patient.

### Detection of carbapenemases

Preliminary screening for carbapenemase activity in *P. aeruginosa* isolate ARGA00461 was performed using cloxacillin inhibition tests (31), the carbapenem inactivation method (CIM) (32) and the β-carba test (Bio-Rad, California, USA). Targeted detection of specific carbapenemase types was subsequently carried out using the OKNVI lateral flow immunoassay (Coris BioConcept, Belgium) and the GeneXpert platform (Cepheid, United Kingdom).

### Resistance genomics

The *P. aeruginosa* ARGA00461 genome was sequenced by short-read Illumina sequencing (Illumina, Inc., California, USA) and long-read PacBio sequencing (Macrogen, South Korea) technologies. Genomic DNA was extracted using the Wizard Genomic DNA Purification Kit (Promega, Wisconsin, USA). Quality control was conducted with fastp (v.0.23.2) (33) for Illumina short reads and filtlong (v.0.2.1) (34) for PacBio long reads, and host contamination was removed with BMTagger (v.3.101) (35). Hybrid genome assembly was performed with Unicycler (v.0.5.0) (36), which was then polished with Polypolish (37). The assembly was then assessed for contamination and completeness with CheckM (v.1.1.3) (38). Putative open reading frames were annotated with Bakta (v1.11.0) (39). The multi-locus sequence type (MLST) was identified with the Center of Genomic epidemiology (CGE)’s MLST tool (40). Horizontally acquired resistome analysis of the strain was conducted with RGI (v.5.2.0) and CARD (v.3.2.8) (41). Chromosomal mutational resistome was further analyzed by variant calling with Snippy (v.4.6.0) (42) and using the genome of *P. aeruginosa* PAO1 (NC_002516.2) as reference. Plasmid detection and prediction of putative mobilization and conjugation capacity were conducted with MOB-suite (v.3.1.0) (43). Plasmid visualizations were created with Brick (v.0.4.1) (44).

### Conjugation experiments

To assess whether the *bla*_OXA-1054_ gene carried on the pOXA-1054 plasmid is transferable to other clinical strains, a filter mating conjugation assay was performed using *P. aeruginosa* ARGA00461 as the donor strain and the rifampicin-resistant *P. aeruginosa* PAO1^Rif^ strain as the recipient (45). Donor and recipient cultures in the logarithmic growth phase were mixed at a 1:1 ratio and applied onto a 0.22 µm membrane filter, followed by overnight incubation at 37°C. After incubation, bacterial suspensions were plated on selective media containing 8 mg/L meropenem and 800 mg/L rifampicin. Putative transconjugants were confirmed by PCR and antimicrobial susceptibility testing. Conjugation efficiency was calculated as the ratio of transconjugants (CFU/mL) to donor cells (CFU/mL).

### Molecular cloning and plasmid manipulation

The *bla*_OXA-1054_ gene was cloned, along with the *bla*_OXA-24_, *bla*_OXA-48_, and *bla*_OXA-198_ genes, into the pUCP24 plasmid and electroporated to the *Escherichia coli* TOP10 reference strain using previously described methodology (46). Transformants were selected on LB agar plates containing 10 mg/L gentamicin. The sequence of the recombinant plasmids was further verified by Sanger sequencing, and the constructs were then electroporated to the *P. aeruginosa* PAO1 reference strain and selected in LB agar plates containing 30 mg/L gentamicin. Moreover, the plasmid pOXA-1054 carrying the *bla*_OXA-1054_ gene harbored by *P. aeruginosa* isolate ARGA00461 was extracted using the GeneJET Plasmid Miniprep Kit (Thermo Fisher, Massachusetts, USA) and transformed into *P. aeruginosa* PAO1 by electroporation. The *P. aeruginosa* PAO1 transformants producing the pOXA-1054 natural plasmid were selected in LB plates containing 10 mg/L meropenem.

### Antimicrobial susceptibility testing

The minimum inhibitory concentrations (MICs) of ticarcillin, piperacillin/tazobactam, aztreonam, aztreonam/avibactam, ceftazidime, ceftazidime/avibactam, ceftolozane/tazobactam, cefepime, cefepime/taniborbactam, cefepime/zidebactam, cefiderocol, imipenem, imipenem/relebactam, meropenem, meropenem/vaborbactam, tobramycin, amikacin, ciprofloxacin and colistin were determined in triplicate by reference broth microdilution assays with cation-adjusted Mueller-Hinton Broth (CAMHB). Iron-depleted CAMHB was used in determining the cefiderocol MICs and prepared according to Clinical and Laboratory Standards Institute (CLSI) M100 guidelines (47). Tazobactam, avibactam, taniborbactam, and relebactam were tested at a fixed concentration of 4 mg/L, while vaborbactam was tested at 8 mg/L. Zidebactam was tested at a 1:1 ratio with cefepime. European Committee on Antimicrobial Susceptibility Testing (EUCAST) v.15.0 clinical breakpoints and guidelines (http://www.eucast.org/clinical_breakpoints/) were applied for interpretation using those of the β-lactam alone for combinations not yet approved. Reference strains *P. aeruginosa* ATCC 27853, *Klebsiella pneumoniae* ATCC 700603, *E. coli* NCTC 13353, and *K. pneumoniae* ATCC BAA-2814 were used as control strains according to the CLSI M100 guidelines.

### Protein purification

The *bla*_OXA-1054_, *bla*_OXA-48_, and *bla*_OXA-198_ genes were cloned into the pGEX-6P-1 plasmid (Cytiva, Massachusetts, USA) using pre-designed primers lacking the signal peptide sequence of the β-lactamases following previously described procedures (48). The recombinant plasmids were transformed into the protease-deficient *E. coli* BL21 strain by electroporation, and transformants were selected in LB plates containing 100 mg/L ampicillin. Protein production was induced using isopropyl β-D-1-thiogalactopyranoside (IPTG), generating glutathione S-transferase (GST) fusion proteins with the OXA enzymes. Fusion proteins (GST/OXA-1054, GST/OXA-48, and GST/OXA-198) were purified to homogeneity with the GST Gene Fusion System (Cytiva, Massachusetts, USA) according to the manufacturer’s instructions. The GST moiety was removed by cleavage with PreScission Protease, and the purity of the final protein extracts (>99%) was ascertained by sodium dodecyl sulfate-polyacrylamide gel electrophoresis (SDS-PAGE).

### Steady-state kinetics

The kinetic parameters of the purified OXA-48, OXA-198, and OXA-1054 β-lactamases for meropenem, imipenem, ceftazidime, ceftolozane, and cefepime were determined as follows: for meropenem and imipenem, the Michaelis-Menten constant *K*_m_ (affinity, antibiotic concentration at which the reaction rate is half of *V*_max_) and *k*_cat_ (turnover rate) values were determined spectrophotometrically in a Specord 200 Plus Spectrophotometer (Analytik Jena, Thuringia, Germany). On the contrary, the *K*_m_ values of ceftazidime, ceftolozane, and cefepime were determined spectrophotometrically as *K*_i app_ with nitrocefin as the reporter substrate in an Epoch 2 Microplate Spectrophotometer (Biotek, Vermont, USA). The *k*_cat_ values were determined by monitoring the direct hydrolysis of the antibiotic at a substrate concentration much higher than the *K*_m_ to ensure the reaction was at *V*_max_. The following wavelengths (λ) and absorption coefficients (ɛ) were used for each antibiotic: λ = 482 nm and ɛ = 15,900 M^−1^ cm^−1^ for nitrocefin, *λ* = 297 nm, and *ɛ* = 10,940 M^−1^ cm^−1^ for meropenem; λ = 297 nm and ɛ = 9,210 M^−1^ cm^−1^ for imipenem; λ = 260 nm and ɛ = 8,660 M^−1^ cm^−1^ for ceftazidime; λ = 254 nm and ɛ = 6,810 M^−1^ cm^−1^ for ceftolozane; and λ = 267 nm and ɛ = 9,120 M^−1^ cm^−1^ for cefepime (48, 49). All parameters were determined in triplicate in 50 mM phosphate (Na_2_HPO_4_) and 20 mM carbonate (NaHCO_3_) buffer, pH 7.2, at room temperature, with standard deviations among replicates below 30% of the mean.

### Relative inhibitory activities of β-lactamase inhibitors

The 50% inhibitory concentration (IC_50_) of tazobactam, avibactam, relebactam, zidebactam, vaborbactam, and taniborbactam against OXA-48, OXA-198, and OXA-1054 was determined spectrophotometrically (Epoch 2 microplate spectrophotometer, Biotek, Vermont, USA) in triplicate experiments with purified enzymes, with nitrocefin as the reporter substrate. The IC_50_ parameter was defined as the inhibitor concentration required to reduce the hydrolysis rate of nitrocefin by 50% following preincubation of the enzyme with different concentrations of each inhibitor at room temperature for 10 min (29).

## Data Availability

The *bla*_OXA-1054_ nucleotide sequence data reported in this work were deposited in the GenBank nucleotide sequence database under accession number PQ507915. The assembly for isolate ARGA00461 (GCA_052906985.1) and its raw reads are available in NCBI under BioProject PRJNA1133624 and associated with BioSample SAMN44420630.

## SUPPLEMENTAL MATERIAL

Supplemental material aac.01805-25-s0001.docx Figs. S1 and S2.

ASM does not own the copyrights to Supplemental Material that may be linked to, or accessed through, an article. The authors have granted ASM a non-exclusive, world-wide license to publish the Supplemental Material files. Please contact the corresponding author directly for reuse.

## References

[1] Horcajada JP, Montero M, Oliver A, Sorlí L, Luque S, Gómez-Zorrilla S, Benito N, Grau S. 2019. Epidemiology and treatment of multidrug-resistant and extensively drug-resistant Pseudomonas aeruginosa infections. Clin Microbiol Rev 32:e00031-19. doi:10.1128/CMR.00031-19 31462403 PMC6730496

[2] Oliver A, Arca-Suárez J, Gomis-Font MA, González-Pinto L, López-Causapé C. 2025. Emerging resistance mechanisms to newer β-lactams in Pseudomonas aeruginosa. Clin Microbiol Infect 31:1790–1796. doi:10.1016/j.cmi.2025.03.013 40120758

[3] Sati H, Carrara E, Savoldi A, Hansen P, Garlasco J, Campagnaro E, Boccia S, Castillo-Polo JA, Magrini E, Garcia-Vello P, et al.. 2025. The WHO bacterial priority pathogens list 2024: a prioritisation study to guide research, development, and public health strategies against antimicrobial resistance. Lancet Infect Dis 25:1033–1043. doi:10.1016/S1473-3099(25)00118-5 40245910 PMC12367593

[4] Candela A, Fernández-Billón M, Aja-Macaya P, González-Pinto L, Fraile-Ribot PA, Viedma E, Alonso-García I, Blanco-Martín T, Estévez-Alfaya R, Fernández-González A, Beceiro A, López-Causapé C, Oviaño M, Bou G, Oliver A, Arca-Suárez J. 2025. Rapid prediction of carbapenemases in Pseudomonas aeruginosa by imipenem/relebactam and MALDI-TOF MS. J Clin Microbiol 63:e0110524. doi:10.1128/jcm.01105-24 40130831 PMC12077086

[5] Sastre-Femenia MÀ, Fernández-Muñoz A, Gomis-Font MA, Taltavull B, López-Causapé C, Arca-Suárez J, Martínez-Martínez L, Cantón R, Larrosa N, Oteo-Iglesias J, Zamorano L, Oliver A, GEMARA-SEIMC/CIBERINFEC Pseudomonas study Group. 2023. Pseudomonas aeruginosa antibiotic susceptibility profiles, genomic epidemiology and resistance mechanisms: a nation-wide five-year time lapse analysis. Lancet Reg Health Eur 34:100736. doi:10.1016/j.lanepe.2023.100736 37753216 PMC10518487

[6] Reyes J, Komarow L, Chen L, Ge L, Hanson BM, Cober E, Herc E, Alenazi T, Kaye KS, Garcia-Diaz J, et al.. 2023. Global epidemiology and clinical outcomes of carbapenem-resistant Pseudomonas aeruginosa and associated carbapenemases (POP): a prospective cohort study. Lancet Microbe 4:e159–e170. doi:10.1016/S2666-5247(22)00329-9 36774938 PMC10016089

[7] González-Pinto L, Alonso-García I, Blanco-Martín T, Camacho-Zamora P, Fraile-Ribot PA, Outeda-García M, Lasarte-Monterrubio C, Guijarro-Sánchez P, Maceiras R, Moya B, Juan C, Vázquez-Ucha JC, Beceiro A, Oliver A, Bou G, Arca-Suárez J. 2024. Impact of chromosomally encoded resistance mechanisms and transferable β-lactamases on the activity of cefiderocol and innovative β-lactam/β-lactamase inhibitor combinations against Pseudomonas aeruginosa. J Antimicrob Chemother 79:2591–2597. doi:10.1093/jac/dkae263 39073766 PMC11441999

[8] Mack AR, Hujer AM, Mojica MF, Taracila MA, Feldgarden M, Haft DH, Klimke W, Prasad AB, Bonomo RA. 2025. β-Lactamase diversity in Pseudomonas aeruginosa. Antimicrob Agents Chemother 69:e0078524. doi:10.1128/aac.00785-24 39927781 PMC11881563

[9] Lasarte-Monterrubio C, Guijarro-Sánchez P, Alonso-Garcia I, Outeda M, Maceiras R, González-Pinto L, Martínez-Guitián M, Fernández-Lozano C, Vázquez-Ucha JC, Bou G, Arca-Suárez J, Beceiro A. 2024. Epidemiology, resistance genomics and susceptibility of Acinetobacter species: results from the 2020 Spanish nationwide surveillance study. Euro Surveill 29:2300352. doi:10.2807/1560-7917.ES.2024.29.15.2300352 38606569 PMC11010588

[10] Poirel L, Bonnin RA, Nordmann P. 2012. Genetic features of the widespread plasmid coding for the carbapenemase OXA-48. Antimicrob Agents Chemother 56:559–562. doi:10.1128/AAC.05289-11 22083465 PMC3256075

[11] Poirel L, Naas T, Nordmann P. 2010. Diversity, epidemiology, and genetics of class D beta-lactamases. Antimicrob Agents Chemother 54:24–38. doi:10.1128/AAC.01512-08 19721065 PMC2798486

[12] El Garch F, Bogaerts P, Bebrone C, Galleni M, Glupczynski Y. 2011. OXA-198, an acquired carbapenem-hydrolyzing class D beta-lactamase from Pseudomonas aeruginosa. Antimicrob Agents Chemother 55:4828–4833. doi:10.1128/AAC.00522-11 21788473 PMC3186976

[13] Lee C-H, Su T-Y, Ye J-J, Hsu P-C, Kuo A-J, Chia J-H, Lee M-H. 2017. Risk factors and clinical significance of bacteremia caused by Pseudomonas aeruginosa resistant only to carbapenems. Journal of Microbiology, Immunology and Infection 50:677–683. doi:10.1016/j.jmii.2015.06.003 26188977

[14] Opperman CJ, Moodley C, Lennard K, Smith M, Ncayiyana J, Vulindlu M, Gafoor M, Govender N, Ismail H, Bamford C, McCarthy KM, Nicol MP, Centner CM. 2022. A citywide, clonal outbreak of Pseudomonas aeruginosa. Int J Infect Dis 117:74–86. doi:10.1016/j.ijid.2022.01.039 35077877

[15] Antonelli A, D’Andrea MM, Vaggelli G, Docquier J-D, Rossolini GM. 2015. OXA-372, a novel carbapenem-hydrolysing class D β-lactamase from a Citrobacter freundii isolated from a hospital wastewater plant. J Antimicrob Chemother 70:2749–2756. doi:10.1093/jac/dkv181 26126492

[16] Bonnin RA, Creton E, Perrin A, Girlich D, Emeraud C, Jousset AB, Duque M, Jacquemin A, Hopkins K, Bogaerts P, Glupczynski Y, Pfennigwerth N, Gniadkowski M, Hendrickx APA, van der Zwaluw K, Apfalter P, Hartl R, Studentova V, Hrabak J, Larrouy-Maumus G, Rocha EPC, Naas T, Dortet L. 2024. Spread of carbapenemase-producing Morganella spp from 2013 to 2021: a comparative genomic study. Lancet Microbe 5:e547–e558. doi:10.1016/S2666-5247(23)00407-X 38677305

[17] Monge-Olivares L, Peñalva G, Pulido MR, Garrudo L, Ángel Doval M, Ballesta S, Merchante N, Rasero P, Cuberos L, Carpes G, López-Cerero L. 2025. Quantitative study of ESBL and carbapenemase producers in wastewater treatment plants in Seville, Spain: a culture-based detection analysis of raw and treated water. Water Res 281:123706. doi:10.1016/j.watres.2025.123706 40311350

[18] Attana F, Kim S, Spencer J, Iorga BI, Docquier J-D, Rossolini GM, Perilli M, Amicosante G, Vila AJ, Vakulenko SB, Mobashery S, Bradford P, Bush K, Partridge SR, Hujer AM, Hujer KM, Bonomo RA, Haider S. 2025. SAND: a comprehensive annotation of class D β-lactamases using structural alignment-based numbering. Antimicrob Agents Chemother Antimicrob Agents Chemother 69:e00150–25. doi:10.1128/aac.00150-25 PMC12217458 40422930

[19] Smith CA, Stasyuk A. 2025. Diversity in the common fold: structural insights into class D β-lactamases from gram-negative pathogens. Pathogens 14:761. doi:10.3390/pathogens14080761 40872271 PMC12389319

[20] Yoon E-J, Jeong SH. 2021. Class D β-lactamases. J Antimicrob Chemother 76:836–864. doi:10.1093/jac/dkaa513 33382875

[21] Savojardo C, Martelli PL, Fariselli P, Casadio R. 2018. DeepSig: deep learning improves signal peptide detection in proteins. Bioinformatics 34:1690–1696. doi:10.1093/bioinformatics/btx818 29280997 PMC5946842

[22] Cao L, Srikumar R, Poole K. 2004. MexAB-OprM hyperexpression in NalC-type multidrug-resistant Pseudomonas aeruginosa: identification and characterization of the nalC gene encoding a repressor of PA3720-PA3719. Mol Microbiol 53:1423–1436. doi:10.1111/j.1365-2958.2004.04210.x 15387820

[23] González-Pinto L, Gomis-Font MA, Pérez-Rodríguez G, Blanco-Martín T, Rodríguez-Pallares S, Sánchez-Peña L, Beceiro A, Bou G, Jeannot K, Oliver A, Arca-Suárez J. 2025. Contribution of mutational resistance mechanisms and acquired β-lactamases to cefiderocol/xeruborbactam susceptibility in Pseudomonas aeruginosa. Antimicrob Agents Chemother 69:e0104825. doi:10.1128/aac.01048-25 41118352 PMC12691625

[24] Kitzinger K, Koch H, Lücker S, Sedlacek CJ, Herbold C, Schwarz J, Daebeler A, Mueller AJ, Lukumbuzya M, Romano S, Leisch N, Karst SM, Kirkegaard R, Albertsen M, Nielsen PH, Wagner M, Daims H. 2018. Characterization of the first “Candidatus Nitrotoga” isolate reveals metabolic versatility and separate evolution of widespread nitrite-oxidizing bacteria. mBio 9:e01186-18. doi:10.1128/mBio.01186-18 29991589 PMC6050957

[25] Lasarte-Monterrubio C, Guijarro-Sánchez P, Bellés A, Vázquez-Ucha JC, Arca-Suárez J, Fernández-Lozano C, Bou G, Beceiro A, Spanish National Study Acinetobacter spp. 2020 Group. 2022. Carbapenem resistance in Acinetobacter nosocomialis and Acinetobacter junii conferred by acquisition of bla_OXA-24/40_ and genetic characterization of the transmission mechanism between Acinetobacter genomic species. Microbiol Spectr 10:e0273421. doi:10.1128/spectrum.02734-21 35138195 PMC8826734

[26] Antunes NT, Lamoureaux TL, Toth M, Stewart NK, Frase H, Vakulenko SB. 2014. Class D β-lactamases: are they all carbapenemases? Antimicrob Agents Chemother 58:2119–2125. doi:10.1128/AAC.02522-13 24468778 PMC4023754

[27] Tsivkovski R, Totrov M, Lomovskaya O. 2020. Biochemical characterization of QPX7728, a new ultrabroad-spectrum beta-lactamase inhibitor of serine and metallo-beta-lactamases. Antimicrob Agents Chemother 64:e00130-20. doi:10.1128/AAC.00130-20 32152086 PMC7269513

[28] Docquier J-D, Calderone V, De Luca F, Benvenuti M, Giuliani F, Bellucci L, Tafi A, Nordmann P, Botta M, Rossolini GM, Mangani S. 2009. Crystal structure of the OXA-48 beta-lactamase reveals mechanistic diversity among class D carbapenemases. Chem Biol 16:540–547. doi:10.1016/j.chembiol.2009.04.010 19477418

[29] Outeda-García M, Arca-Suárez J, Lence E, Rodriguez-Coello A, Maceiras R, Blanco-Martin T, Guijarro-Sánchez P, Gonzalez-Pinto L, Alonso-Garcia I, García-Pose A, Muras A, Rodriguez-Pallares S, Lasarte-Monterrubio C, Gonzalez-Bello C, Vázquez-Ucha JC, Bou G, Beceiro A. 2025. Advancements in the fight against globally distributed OXA-48 carbapenemase: evaluating the new generation of carbapenemase inhibitors. Antimicrob Agents Chemother 69:e0161424. doi:10.1128/aac.01614-24 39791889 PMC11823609

[30] Mojica MF, Zeiser ET, Becka SA, Six DA, Moeck G, Papp-Wallace KM. 2024. Cefepime-taniborbactam demonstrates potent in vitro activity vs Enterobacterales with bla OXA-48. Microbiol Spectr 12. doi:10.1128/spectrum.01144-24 PMC11537129 39315842

[31] Fournier D, Garnier P, Jeannot K, Mille A, Gomez A-S, Plésiat P. 2013. A convenient method to screen for carbapenemase-producing Pseudomonas aeruginosa. J Clin Microbiol 51:3846–3848. doi:10.1128/JCM.01299-13 23966511 PMC3889778

[32] van der Zwaluw K, de Haan A, Pluister GN, Bootsma HJ, de Neeling AJ, Schouls LM. 2015. The carbapenem inactivation method (CIM), a simple and low-cost alternative for the Carba NP test to assess phenotypic carbapenemase activity in gram-negative rods. PLoS One 10:e0123690. doi:10.1371/journal.pone.0123690 25798828 PMC4370852

[33] Chen S, Zhou Y, Chen Y, Gu J. 2018. Fastp: an ultra-fast all-in-one FASTQ preprocessor. Bioinformatics 34:i884–i890. doi:10.1093/bioinformatics/bty560 30423086 PMC6129281

[34] Wick RR, Menzel P. 2017. Filtlong. Available from: https://github.com/rrwick/Filtlong

[35] Rotmistrovsky K, Agarwala R. 2011. BMTagger: Best Match Tagger for removing human reads from metagenomics datasets. NCBI/NLM, National Institutes of Health 2011

[36] Wick RR, Judd LM, Gorrie CL, Holt KE. 2017. Unicycler: resolving bacterial genome assemblies from short and long sequencing reads. PLoS Comput Biol 13:e1005595. doi:10.1371/journal.pcbi.1005595 28594827 PMC5481147

[37] Wick RR, Holt KE. 2022. Polypolish: short-read polishing of long-read bacterial genome assemblies. PLoS Comput Biol 18:e1009802. doi:10.1371/journal.pcbi.1009802 35073327 PMC8812927

[38] Parks DH, Imelfort M, Skennerton CT, Hugenholtz P, Tyson GW. 2015. CheckM: assessing the quality of microbial genomes recovered from isolates, single cells, and metagenomes. Genome Res 25:1043–1055. doi:10.1101/gr.186072.114 25977477 PMC4484387

[39] Schwengers O, Jelonek L, Dieckmann MA, Beyvers S, Blom J, Goesmann A. 2021. Bakta: rapid and standardized annotation of bacterial genomes via alignment-free sequence identification. Microb Genom 7:000685. doi:10.1099/mgen.0.000685 34739369 PMC8743544

[40] Larsen MV, Cosentino S, Rasmussen S, Friis C, Hasman H, Marvig RL, Jelsbak L, Sicheritz-Pontén T, Ussery DW, Aarestrup FM, Lund O. 2012. Multilocus sequence typing of total-genome-sequenced bacteria. J Clin Microbiol 50:1355–1361. doi:10.1128/JCM.06094-11 22238442 PMC3318499

[41] Alcock BP, Huynh W, Chalil R, Smith KW, Raphenya AR, Wlodarski MA, Edalatmand A, Petkau A, Syed SA, Tsang KK, et al.. 2023. CARD 2023: expanded curation, support for machine learning, and resistome prediction at the Comprehensive Antibiotic Resistance Database. Nucleic Acids Res 51:D690–D699. doi:10.1093/nar/gkac920 36263822 PMC9825576

[42] Seemann T. 2015. Snippy: fast bacterial variant calling from NGS reads. https://github.com/ tseemann/snippy.

[43] Robertson J, Nash JHE. 2018. MOB-suite: software tools for clustering, reconstruction and typing of plasmids from draft assemblies. Microb Genom 4:e000206. doi:10.1099/mgen.0.000206 30052170 PMC6159552

[44] Steinig E. 2024. BRIG-like interactive data visualization for prokaryotic genome annotation, comparison and exploration of large-scale genomic regions. https://github.com/esteinig/brick.

[45] Zhou L, Yang C, Zhang X, Yao J, Chen L, Tu Y, Li X. 2023. Characterization of a novel Tn6485h transposon carrying both blaIMP-45 and blaAFM-1 integrated into the IncP-2 plasmid in a carbapenem-resistant Pseudomonas aeruginosa. J Glob Antimicrob Resist 35:307–313. doi:10.1016/j.jgar.2023.10.010 37879457

[46] Blanco-Martín T, Alonso-García I, González-Pinto L, Outeda-García M, Guijarro-Sánchez P, López-Hernández I, Pérez-Vázquez M, Aracil B, López-Cerero L, Fraile-Ribot P, Oliver A, Vázquez-Ucha JC, Beceiro A, Bou G, Arca-Suárez J, GEMARA/SEIMC-CIBERINFEC Study Group on the activity and resistance mechanisms to new β-lactams and β-lactamase inhibitors (PROTECT). 2024. Activity of cefiderocol and innovative β-lactam/β-lactamase inhibitor combinations against isogenic strains of Escherichia coli expressing single and double β-lactamases under high and low permeability conditions. Int J Antimicrob Agents 63:107150. doi:10.1016/j.ijantimicag.2024.107150 38513748

[47] CLSI. 2020. Performance standards for antimicrobial susceptibility testing. 30th edition. CLSI supplement M100. Clinical and Laboratory Standards Institute

[48] González-Pinto L, Gomis-Font MA, Lence E, Outeda-García M, Blanco-Martín T, Rodríguez-Pallares S, Sánchez-Peña L, Alonso-García I, Vázquez-Ucha JC, Beceiro A, Bou G, González-Bello C, Oliver A, Arca-Suárez J. 2025. Functional and structural analyses of amino acid sequence variation in PDC β-lactamase reveal different mechanistic pathways toward cefiderocol resistance in Pseudomonas aeruginosa. Antimicrob Agents Chemother 69:e0029225. doi:10.1128/aac.00292-25 40422084 PMC12217463

[49] Arca-Suárez J, Fraile-Ribot P, Vázquez-Ucha JC, Cabot G, Martínez-Guitián M, Lence E, González-Bello C, Beceiro A, Rodríguez-Iglesias M, Galán-Sánchez F, Bou G, Oliver A. 2019. Challenging antimicrobial susceptibility and evolution of resistance (OXA-681) during treatment of a long-term nosocomial infection caused by a Pseudomonas aeruginosa ST175 clone. Antimicrob Agents Chemother 63:e01110–19. doi:10.1128/AAC.01110-19 PMC6761516 31383659

