# Supplementary material for: Genetic and biochemical characterization of OXA-1054, a carbapenem-hydrolyzing class D β-lactamase conferring broad-spectrum β-lactam resistance in Pseudomonas aeruginosa
Source: Antimicrob Agents Chemother. 2026 May 29;70(7):e01805-25. doi: 10.1128/aac.01805-25 (PMC13321841; doi:10.1128/aac.01805-25)
Supplement: Supplemental material — Figs. S1 and S2. [file aac.01805-25-s0001.docx]

**
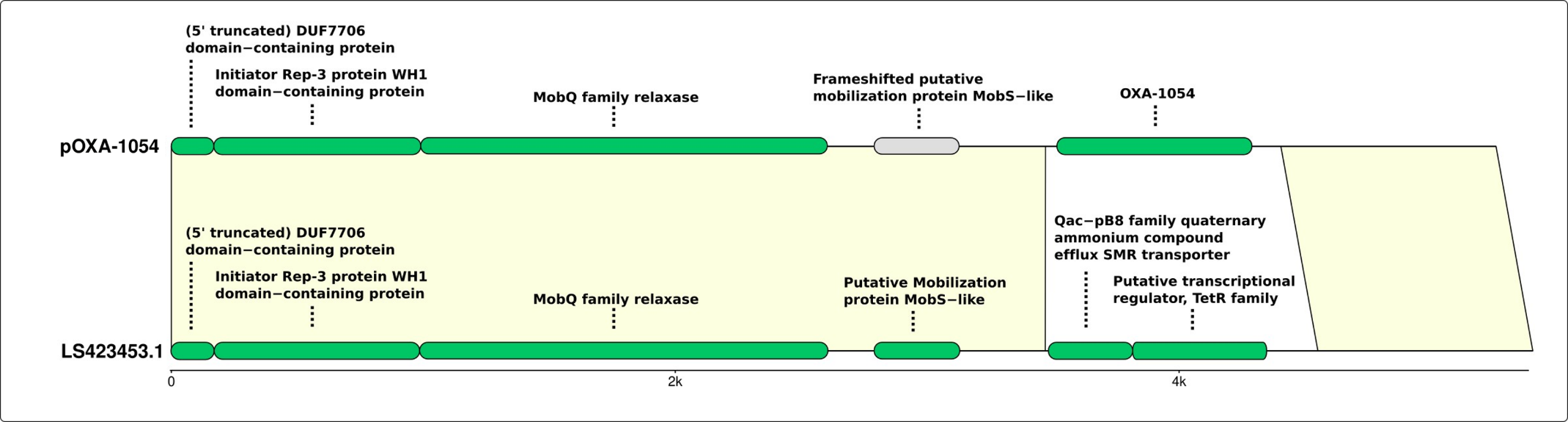
**

**FIGURE S1**: Comparative genomic analysis of plasmid pOXA-1054 and the *Candidatus* *Nitrotoga fabula* plasmid (LS423453.1). Yellow-shaded regions illustrate the 83% sequence conservation between the two plasmids. Green boxes represent predicted open reading frames (ORFs). Key genetic divergences are highlighted: (i) the MobS-like mobilization protein is truncated in pOXA-1054 (grey box) compared to the intact version in LS423453.1; (ii) the *bla*_OXA-1054_ carbapenemase gene in pOXA-1054 is replaced in the *Candidatus Nitrotoga fabula* plasmid by a Qac-pB8 family SMR efflux transporter and a TetR-family transcriptional regulator.


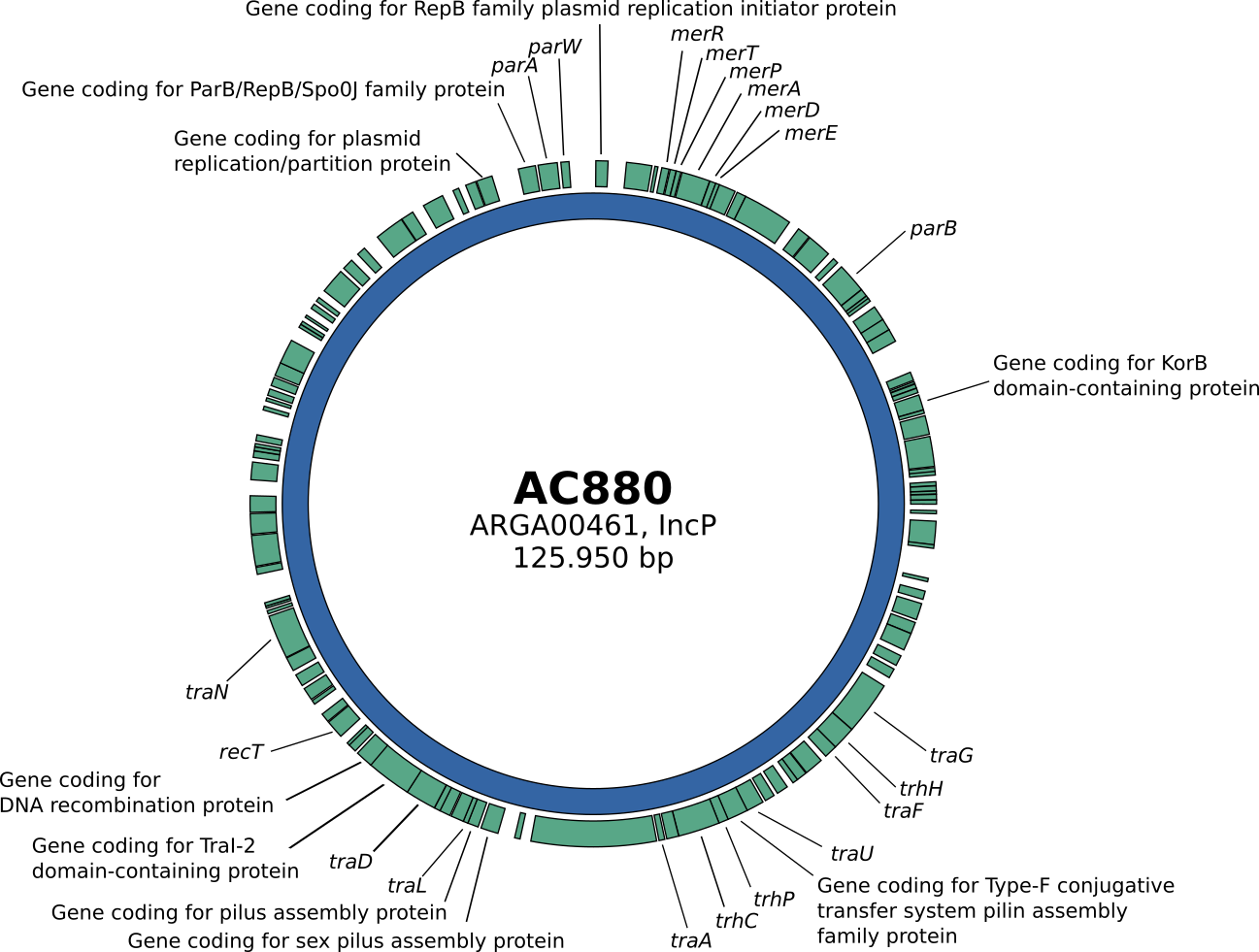


**FIGURE S2**: Genetic context of the pAC880 plasmid harbored by *P. aeruginosa* isolate ARGA00461.
